# Curriculum Innovations: Implementation of a Targeted Longitudinal Rotation to Enhance Direct Ophthalmoscopy Learning for Neurology Residents

**DOI:** 10.1212/NE9.0000000000200242

**Published:** 2025-09-05

**Authors:** Noor Chahal, Maria Lee, Emily Eng, Thanh-Liem Huynh-Tran, Jenny Ji-hyun Lee, Nailyn Rasool, Mark Terrelonge, Laura Bonelli, Tania Onclinx, Alice Jiang, Madeline Yung

**Affiliations:** 1 Department of Ophthalmology, University of California, San Francisco;; 2 Department of Optometry, University of California, San Francisco;; 3 Department of Neurology, University of California, Los Angeles;; 4 Department of Neurology, Department of Ophthalmology, University of California, San Francisco;; 5 Department of Neurology, University of California, San Francisco;; 6 Department of Neurology, Department of Ophthalmology, University of California, Los Angeles; and; 7 Department of Ophthalmology, University of California, Los Angeles.

## Abstract

**Background and Objectives:**

Direct ophthalmoscopy (DO) is a core diagnostic skill for neurology residents. Although the importance of DO is accepted among neurology faculty and residents, proficiency in DO has declined because of limited training opportunities. The objectives of this study were to (1) provide neurology residents with supervised and unsupervised DO practice, (2) improve their competency and confidence in performing DO, and (3) develop a curriculum model adaptable to residencies with limited neuro-ophthalmology exposure.

**Methods:**

A longitudinal, practice-based optometry or ophthalmology elective was implemented at 2 neurology residency programs, guided by Kern's 6-step approach to curriculum development. The rotation included both supervised and graduated independent practice. Residents completed anonymized pre-rotation and post-rotation questionnaires measuring confidence in performing DO and underwent objective skills assessments to measure competence in DO.

**Results:**

A total of 13 neurology residents in postgraduate years 2–4 participated in the elective for an average of 4.8 half-days per resident over the course of the academic year. Residents demonstrated a significant increase in the number of ocular examinations performed by the end of the rotation. On completion of the elective, residents reported a mean increase in confidence (Likert scale 1–5) in focusing on the retina (+2.0 [95% CI 1.3–2.7], *p* = 0.005), finding the optic disc (+1.7 [0.9–2.6], *p* = 0.013), finding retinal blood vessels (+1.8 [1.1–2.5], *p* = 0.008), and recognizing optic disc pathology (+1.4 [0.7–2.1], *p* = 0.013). Mean DO objective assessment scores improved by 46.6% [27.8%–64.7%] (*p* = 0.006). Residents who performed 50 or more undilated DO examinations and those who were confident in finding the optic disc were more likely to incorporate DO into their clinical practice.

**Discussion:**

Integrating an optometry or ophthalmology rotation into neurology residencies enhanced both subjective confidence and objective competence in DO, underscoring the importance of deliberate, structured practice in mastering diagnostic examination skills. Incorporation of self-regulated learning techniques and user-friendly technology may further enhance curricular efficacy. These findings demonstrate the feasibility of adopting this curriculum across various residency programs, particularly those that recognize the value of fundoscopic examination but lack adequate training opportunities.

## Introduction

Direct ophthalmoscopy (DO) is a fundamental clinical skill that allows clinicians to noninvasively examine the optic nerve, retina, and posterior ocular structures to diagnose neurologic, ophthalmic, and systemic conditions.^1^ It is often indicated in the assessment of patients with headache, eye pain, or vision loss and can offer valuable insights into the diagnosis and management of various vision or life-threatening neurologic conditions, such as in cases of optic neuritis and ischemic optic neuropathy.^2^ In addition, a thorough examination of the ocular fundus is crucial for evaluating neurologic disorders because some conditions may only be detectable through changes in the ocular fundus, even if other clinical signs are absent.^3^ Although previous studies have identified fundus photography as a useful tool for ocular screening, DO remains valuable because of its accessibility, affordability, portability, rapid assessment time, and strong accuracy in identifying serious ocular conditions.^4-6^

However, proficiency in performing DO has declined among nonophthalmologists in recent decades.^3-8^ Achieving competency in DO remains a challenge for graduate medical trainees, including neurology residents, in part because of a lack of targeted educational interventions or structured curricula.^9^ A recent needs assessment found that 35.7% of graduating neurology residents never correctly identified an abnormal finding on DO during residency.^9^ Although various educational strategies such as simulations, smartphone attachments, and fundus imaging have been explored in undergraduate medical education, there has been limited research and guidance for DO curricula in graduate medical education.^9-15^ The American Board of Psychiatry and Neurology's core competencies and the Accreditation Council for Graduate Medical Education's neurology program requirements emphasize the importance of the neuro-ophthalmologic examination, but specific elements such as DO are not explicitly delineated.^16,17^ This gap in targeted DO education results in underprepared physicians who are at higher risk of missing or delaying diagnoses, potentially compromising patient care.^18-21^ Addressing these deficiencies in graduate medical training by developing innovative educational strategies is a critical priority.

To design and implement our curricular innovation, we followed Kern's 6-step approach to curriculum development.^22^ Our recent nationwide needs assessment revealed a strong motivation among neurology program directors (PDs) and residents to enhance DO education.^9^ Primary barriers to learning included deficient opportunities for supervised practice and a lack of structured time for learning. Practice skills sessions and supervised practice were identified as the 2 most helpful educational interventions. Although a large proportion of neurology residents ranked “rotations through ophthalmology” as a “most helpful” intervention for learning DO, only 5% of programs had a required neuro-ophthalmology rotation.^9^ To increase educational opportunities for DO, we explored rotations in nontraditional clinical settings. We thus designed an optometry/general ophthalmology–based elective for neurology residents to provide dedicated learning time with access to pathology and supervised practice while addressing the limited educational access to neuro-ophthalmology. We report initial results from a pilot of this new curriculum regarding its feasibility, impact on DO competency, and satisfaction with DO training.

### Objectives

1. To provide residents with opportunities for supervised and unsupervised DO practice.
2. To improve residents' technical competency and confidence in performing DO.
3. To outline a curricular model that can be expanded to neurology residencies with low neuro-ophthalmology exposure and to other nonophthalmology training programs that frequently care for patients requiring a fundus examination (i.e., emergency medicine, primary care, etc.).

## Methods

### Curriculum Participants

The optometry (University of California, Los Angeles, UCSF) or ophthalmology (University of California, Los Angeles, UCLA) rotation was offered as a neurology residency elective to all interested neurology residents in postgraduate years (PGYs) 2–4 at UCSF and UCLA during the 2023–2024 academic year. Preceptors for the curriculum consisted of a neuro-ophthalmology–trained optometrist (EE), a comprehensive ophthalmologist (TO), and a neuro-ophthalmologist (LB).

### Curriculum Structure

Kern's 6-step approach to curriculum development was used to guide the creation of this rotation for neurology residents.^22^ As stated above, a review of the literature highlighted a decline in competency among nonophthalmologists in performing DO (Step 1: Problem Identification and Needs Assessment), and we conducted a nationwide needs assessment to gather perspectives from neurology PDs and residents regarding DO (Step 2: Targeted Needs Assessment).^9^ The assessment revealed a significant gap: although the importance of DO was recognized, there was a significant disparity between the number of DO examinations expected by PDs and the number actually performed by residents. To address this gap, we developed a curriculum to provide more opportunities for DO practice, designed to improve residents' competence and confidence in DO (Step 3: Goals and Objectives).

The curriculum consisted of a longitudinal elective, where residents participated in 3–7 half-day clinic sessions in optometry (UCSF) or comprehensive ophthalmology/neuro-ophthalmology clinics (UCLA) over the course of the academic year. This elective allowed residents to practice DO and receive targeted feedback from experts in fundoscopy (Step 4: Educational Strategies). Equipment used to perform DO was dependent on availability: UCSF residents used conventional direct ophthalmoscopes (*Coaxial, Welch Allyn, Skaneateles Falls, NY*) while UCLA residents used PanOptic direct ophthalmoscopes (*PanOptic, Welch Allyn, Skaneateles Falls, NY*).

### Curriculum Implementation

At UCSF, neurology residents participated in half-day optometry clinic sessions led by a neuro-ophthalmology-trained optometrist. At UCLA, rotations included half-day sessions with a comprehensive ophthalmologist (Tania Onclinx) TO in an urgent care setting and a neuro-ophthalmologist (Laura Bonelli) in an outpatient neuro-ophthalmology clinic; residents were assigned to a combination of both clinics based on the availability of the resident and the attending. At both institutions, sessions were conducted over the course of a full year, and residents were expected to independently complete full clinical encounters, including history-taking, ophthalmic examinations, and management planning, with graded independence and feedback (Step 5: Implementation).

The workflow at both sites followed a consistent structure. Regardless of the chief concern, residents conducted DO on each patient. For solely ocular surface issues, only undilated examinations were performed. For patients requiring dilation, such as those reporting flashes or floaters, residents completed the DO examination twice—once before and once after dilation to confirm their findings. Each resident's observations, such as cup-to-disc ratio, disc margins, and macula findings, were verified by preceptors using either an ophthalmoscope or a slit lamp. The initial DO examination was fully supervised to assess baseline skills, with real-time and/or postvisit feedback provided by preceptors. Subsequent examinations were performed independently, with feedback focused on examination findings.

### Curriculum Assessment

Guided by the Kirkpatrick model, we used a combination of quantitative and qualitative methods to evaluate our curriculum's primary outcomes, focusing on residents' reactions (Kirkpatrick level 1) and their learning and skill acquisition through self-reported confidence and preceptor-conducted objective assessments (Kirkpatrick level 2).^23^

We used anonymized pre-rotation and post-rotation questionnaires modified from a previously validated questionnaire.^24-27^ To maintain anonymity, participants were asked to create unique identifiers, instead of their names, to link the questionnaires. These questionnaires were administered electronically through REDCap (Nashville, TN) at UCSF and Qualtrics (Provo, UT) at UCLA. The survey content was identical across both institutions. The questionnaires assessed participants' experiences and comfort with various eye examinations, their confidence in performing DO, and their satisfaction with the DO training provided during residency. The questionnaires used a 5-point Likert scale and included free-text options to denote the number of examinations performed and qualitative feedback for the curriculum (Step 6: Evaluation and Feedback). In addition, we included questions to explore the potential long-term effects of the curriculum, such as the likelihood of residents self-initiating DO in appropriate clinical situations and incorporating DO into their clinical practice after graduation. The questionnaires were reviewed by an AssociatePD for the UCSF Neurology residency (Mark Terrelonge), the Division Chief of Neuro-Ophthalmology at UCSF (Nailyn Rasool), and the UCSF Center for Faculty Educators.

For objective assessments, we adopted a previously validated tool from an Objective Structured Clinical Examination used to evaluate medical students and ophthalmology residents, where higher scores correlated with improved ability to identify retinal pathology.^28^ Each assessment form consisted of a 15-item technique checklist evaluating both examination technique and fundus visualization, with points ranging from 1 to 3 for each item. Points were awarded for each component in an all-or-nothing fashion with a maximum possible total of 23 points. Preceptors completed these assessments in real time while observing residents perform DO on patients in clinic. The pre-rotation assessment was performed on the first patient seen on the first day of the residents' rotation and the post-rotation assessment on the last patient seen on the last day. We initially attempted assessments on undilated eyes; however, owing to low success rates, the protocol was adjusted to perform all assessments on dilated eyes for standardization.

Post-rotation data were compared with pre-rotation data, and statistical analysis was conducted using the Wilcoxon signed-rank test for continuous variables and the Fisher exact test for categorical variables. A Mann-Whitney *U* test was used for further stratified analysis. Pearson correlation analysis was used to evaluate the relationship between technique and fundus visualization scores. A *p* value <0.05 was used to indicate significance. Responses to Likert scale questions were categorized to reflect overall sentiment. For questions related to confidence, the 2 positive choices (“very confident” and “somewhat confident”) were aggregated to denote “confident” residents while the remaining choices (“very unconfident,” “somewhat unconfident,” and “neutral”) were aggregated to denote “unconfident” residents. Responses for likelihood, comfort, and satisfaction were grouped in a similar manner. All questionnaires and assessments are available in the supplemental material.

### Standard Protocol Approvals, Registrations, and Participant Consents

This study was reviewed by the Institutional Review Boards of the University of California, San Francisco (UCSF; IRB#22-3677), and the University of California, Los Angeles (UCLA; IRB#23-000384), and deemed IRB exempt. Participation in the questionnaires and objective assessments was voluntary. Consent for participation in the surveys was provided on the first page of each questionnaire. Participants were considered to have given implied consent by continuing to complete the survey.

### Data Availability

Anonymized data not published within this article will be made available by request from any qualified investigator.

## Results

### Questionnaire and Assessment Completion Rates

Thirteen neurology residents (9 from UCSF and 4 from UCLA) enrolled in the longitudinal optometry or ophthalmology elective. Twelve residents (92.3%) completed the pre-rotation questionnaire, and all 13 residents (100%) completed the pre-rotation objective assessment. Ten residents (76.9%) submitted the post-rotation questionnaire, and 11 residents (84.6%) participated in the post-rotation DO objective assessment.

For the purposes of this curricular evaluation, data analysis was performed using only those residents who completed both pre-rotation and post-rotation questionnaires and objective assessments. Specifically, this included 10 residents (6 from UCSF and 4 from UCLA) for the questionnaires and 11 residents (7 from UCSF and 4 from UCLA) for the objective assessments.

### Resident Characteristics

The average age of the residents who responded to the questionnaire was 30.3 ± 2.5 years (mean ± SD). Four residents (40.0%) were male, and 6 residents (60.0%) were female. The distribution of residents by year of training was as follows: PGY2 (n = 1, 10.0%), PGY3 (n = 7, 70.0%), and PGY4 (n = 2, 20.0%). The residents' intended subspecialties after graduation were as follows: neurohospitalist (n = 2, 20.0%), neurocritical care (n = 2, 20.0%), movement disorders (n = 2, 20.0%), stroke (n = 1, 10.0%), headache (n = 1, 10.0%), palliative care (n = 1, 10.0%), and none (n = 1, 10.0%).

### Curriculum Participation

The average number of half-day sessions attended by the neurology residents in the optometry or ophthalmology rotation was 4.8 ± 1.2. UCLA residents attended 5.8 ± 0.5 half-day sessions, and UCSF residents attended 4.3 ± 1.1 half-day sessions.

### Results

**Figure 1:**
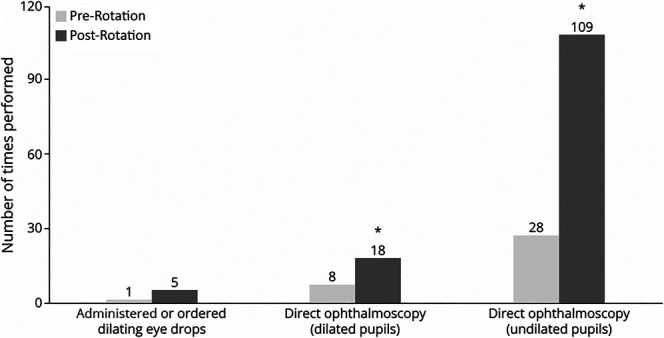
Performance of Ocular Examination Components The average number of times residents performed the following skills: administering or ordering dilating eye drops, direct ophthalmoscopy on dilated pupils, and direct ophthalmoscopy on undilated pupils. Asterisk denotes *p* < 0.05 using the Wilcoxon signed-rank test.

Residents gained experience across all ocular examination components, with notable increases in patient volume, especially for undilated examinations, while experience with dilated examinations and administering dilating drops remained more limited (Figure 1; eFigure 1).

**Figure 2:**
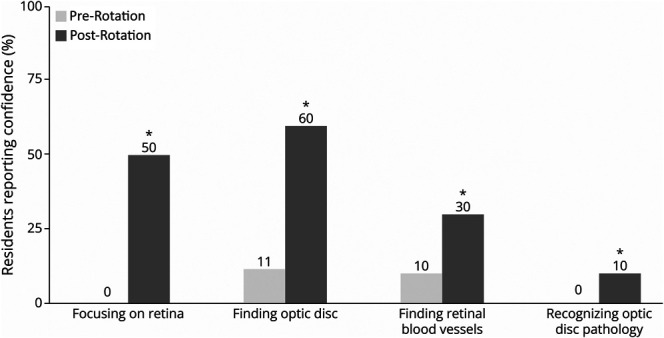
Resident Confidence in Direct Ophthalmoscopy Components Residents reporting “very confident” or “somewhat confident” in different components of the direct ophthalmoscopy examination before rotation and after rotation. Asterisk denotes *p* < 0.05 using the Wilcoxon signed-rank test.

Before the rotation, 0.0% of residents reported overall confidence (responses of 4 [somewhat confident] or 5 [very confident] on a 5-point Likert scale) in performing DO for both dilated and undilated pupils. After the rotation, these percentages increased to 50.0% for dilated pupils and 20.0% for undilated pupils, with mean confidence scores increasing by 1.8 [95% CI 1.1–2.5] (*p* = 0.008) and 1.1 [0.4–1.8] (*p* = 0.020), respectively (Kirkpatrick level 2).^23^ Before the rotation, residents estimated that they could successfully visualize the optic nerve on DO in 20.1% of undilated patients. This estimate significantly increased to 48.1% by the end of the rotation, a mean increase of 28.0% [11.6%–44.4%] (*p* = 0.016). In addition to overall confidence, residents' confidence in the specific components of DO also increased significantly: focusing on the retina (2.0 [1.3–2.7], *p* = 0.005), finding the optic disc (1.7 [0.9–2.6], *p* = 0.013), finding retinal blood vessels (1.8 [1.1–2.5], *p* = 0.008), and recognizing optic disc pathology (1.4 [0.7–2.1], *p* = 0.013) (Figure 2).

**Figure 3:**
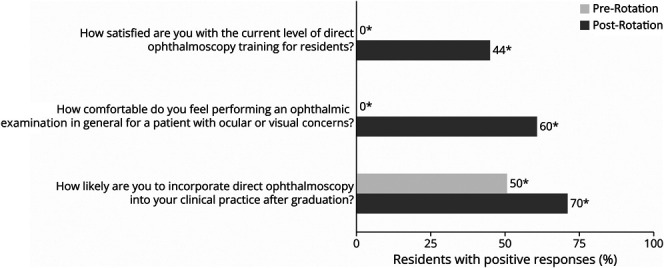
Resident Survey Responses Comparison of residents' positive responses before rotation and after rotation. Positive responses reflect the 2 positive choices on the 5-point Likert scale, denoted by a response of “4” or “5” on the Likert scale. Asterisk denotes *p* < 0.05 using the Wilcoxon signed-rank test.

After the rotation, residents reported significantly greater likelihood to incorporate DO into practice (1.1 [0.3–1.9], *p* = 0.024), increased comfort with ophthalmic examinations (1.6 [0.6–2.6], *p* = 0.013), and higher satisfaction with training (1.7 [0.6–2.7], *p* = 0.021) (Figure 3). The stratified analysis revealed that 100.0% [47.8%–100.0%] of residents who administered or ordered dilating eye drops for 1 or more patients (n = 5) intended to incorporate DO into their clinical practice, compared with 40.0% [5.3%–85.3%] of those who did not administer or order any dilating eye drops (n = 5) (*p* = 0.015). Residents who felt confident in locating the optic disc after the rotation (n = 6) were significantly more likely to incorporate DO into their clinical practice (100.0% [54.1%–100.0%]) compared with those who were not confident (n = 4) (25.0% [0.6%–80.6%], *p* = 0.018). Similarly, residents who performed 50 or more undilated DO examinations by the end of the rotation (n = 5) reported a significantly higher intention to incorporate DO into their clinical practice (100.0% [47.8%–100.0%]) compared with those who performed fewer than 50 undilated examinations (40.0% [5.3%–85.3%], *p* = 0.015). There was no significant association between the number of dilated DO examinations and their likelihood of self-initiating DO, likelihood of incorporating DO into clinical practice, comfort performing the ophthalmic examination, or satisfaction with the current level of DO training.

After completing the rotation, 88.9% of residents felt that it was helpful in improving their confidence in performing DO. Likewise, 88.9% indicated that they would recommend the rotation to other neurology residents (Kirkpatrick level 1).^23^

### Objective Assessment Results

**Figure 4:**
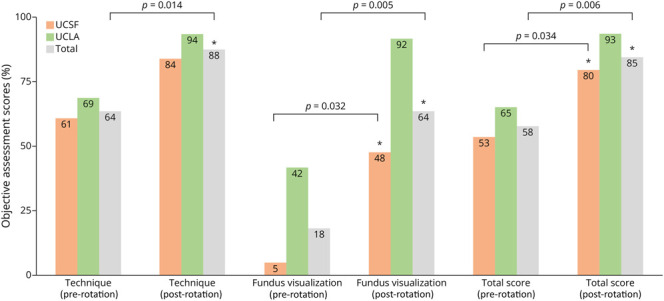
Mean Percent Scores on Objective Assessment Objective assessment scores were divided into technique (maximum score: 20.0) and fundus visualization (maximum score: 3.0) components, presented separately for UCSF, UCLA, and total participants. Significance for UCLA residents' scores could not be established because of sample size limitations (n = 4). Asterisk denotes *p* < 0.05 using the Wilcoxon signed-rank test.

Residents demonstrated significant improvements in both the technique and fundus visualization portions of the 23-point DO objective assessment, with total scores increasing from 13.3 ± 4.2 to 19.5 ± 2.4, a mean increase of 6.2 [3.7–8.6] (*p* = 0.006) by the end of the rotation (Figure 4). Specifically, the percentage of residents who could successfully focus the ophthalmoscope increased from 18.2% [2.2%–51.8%] before rotation to 72.7% [39.0%–94.0%] after rotation (*p* = 0.030). There was also a notable increase in residents' ability to measure the cup-to-disc ratio within 0.1 of the preceptor's measurement, from 0.0% [0.0%–28.5%] before the rotation to 54.5% [23.4%–83.3%] afterward (*p* = 0.012). In addition, 100.0% [71.5%–100.0%] of residents were able to identify disc margins by the end of the rotation compared with 45.5% [16.8%–76.6%] before rotation (*p* = 0.012) (Kirkpatrick level 2).^19^ There was a significant positive correlation between technique and fundus visualization scores (*r* = 0.75, *r*^2^ = 0.56, *p* = 0.008). On stratified analysis, there was no association between questionnaire responses and objective assessment scores.

### Comparing Results Between Institutions

Owing to the small sample size of UCLA residents (n = 4), the parameters for the Mann-Whitney *U* test were not met. As such, institutional data are presented descriptively.

All 4 UCLA residents (100.0%) submitted both the pre-rotation and post-rotation questionnaires and the pre-rotation and post-rotation objective assessments. Of the 9 UCSF residents, 8 (88.9%) completed the pre-rotation questionnaire and all 9 residents (100.0%) completed the pre-rotation objective assessment. Six UCSF residents (66.7%) completed the post-rotation questionnaire, and 7 (77.8%) completed the post-rotation objective assessment. The average postgraduate year of residency was PGY 3.2 ± 0.8 for UCSF residents and PGY 3.0 ± 0.0 for UCLA residents.

The average total pre-rotation objective assessment score was 12.3 ± 4.2 for UCSF residents and 15.0 ± 4.2 for UCLA residents. Post-rotation assessments showed improvements in both objective and subjective measures at both institutions (Table). After rotation, UCSF residents' objective assessment scores increased to 18.3 ± 2.2 and UCLA residents' scores increased to 21.5 ± 0.6 (Figure 4).

**Table:** Comparison of Subjective and Objective Pre-Rotation and Post-Rotation Metrics by Institution

	UCSF	UCLA	UCSF	UCLA
	Pre-rotation (N = 7)	Pre-rotation (N = 4)	Post-rotation (N = 7)	Post-rotation (N = 4)
Average confidence in checking visual acuity	2.8 ± 1.0	4.0 ± 0.0	4.3 ± 0.8	4.8 ± 0.5
Average comfort in performing ophthalmic examination for patients with ocular/visual concerns	2.5 ± 0.5	1.5 ± 0.6	3.5 ± 1.0	4.0 ± 0.8
Average likelihood of self-initiating direct ophthalmoscopy while evaluating a patient who needs it	3.3 ± 1.4	2.8 ± 0.5	3.8 ± 0.8	4.3 ± 1.0
Percentage who checked beam size and brightness (%)	100.0	25.0	100.0	100.0
Percentage who identified optic disc margins (%)	14.3	100.0	100.0	100.0
Percentage who measured a cup-to-disc ratio within 0.1 of the preceptor's measurement (%)	0.0	0.0	28.6	100.0
Percentage who identified the macula (%)	0.0	25.0	14.3	75.0
Average total objective assessment score	12.3 ± 4.2	15.0 ± 4.2	18.3 ± 2.2	21.5 ± 0.6

Abbreviations: UCLA = University of California, Los Angeles; UCSF = University of California, San Francisco.

Data are presented as mean ± SD for Likert scale measures, percentages for objective assessment components, and mean ± SD for average objective assessment scores. The total possible score for the objective assessment was 23.0.

## Discussion

We implemented a longitudinal, practice-based outpatient optometry and ophthalmology rotation that successfully achieved several key outcomes: (1) providing neurology residents with opportunities for both supervised and independent DO practice, (2) enhancing DO competency and confidence, and (3) modeling a curriculum that can be adapted to residencies with limited neuro-ophthalmology exposure. Our curriculum achieved both Kirkpatrick level 1 and level 2 outcomes, as residents demonstrated meaningful improvements in technical skills and increased confidence across core components of the DO examination.^23^ This study serves as a proof of concept that optometry or general ophthalmology clinics can provide neurology residents with valuable hands-on training in essential examination skills, helping address critical gaps in residency education. Furthermore, given the national shortage of neuro-ophthalmologists, this curriculum may also increase resident exposure and foster interest in neuro-ophthalmology as a future career path.^29^

Residents who performed 50 or more undilated DO examinations, ordered/administered dilating eye drops at least once, or reported confidence in finding the optic disc were significantly more likely to intend to incorporate DO into their clinical practice after graduation. This suggests that increasing hands-on practice may directly translate to future clinical behavior, as learners with more experience demonstrated a greater commitment to using these skills after their training. Of interest, administering dilation drops may present a promising avenue for increasing DO practice among general providers, particularly in nonophthalmology settings where dilation is not commonly performed.^30^ Dilation simplifies DO technique by improving the view of the retina, which may encourage nonophthalmologists to actively learn and practice this diagnostic skill.^31^ While this study highlights the importance of deliberate practice in building DO competence and confidence, the underlying mechanisms of self-regulated learning for DO are not delineated and warrant further investigation.

The post-rotation assessment revealed that residents showed the greatest improvement in technical skills such as focusing the ophthalmoscope, identifying disc margins, and obtaining an accurate cup-to-disc ratio. Although there was also a significant increase in confidence in recognizing optic disc pathology, these interpretive gains were more modest (Figure 2). This may reflect both limited exposure to pathology during clinic sessions and the early stage of learners' skill development. It is important to note that we observed a strong positive correlation between technique and fundus visualization scores (*r* = 0.75, *p* = 0.008), reinforcing the principle that reliable visualization is a necessary precursor to meaningful interpretation. For this reason, our curriculum intentionally prioritized technical proficiency as a foundational step before placing formal emphasis on diagnostic interpretation. From a neurology perspective, key fundus findings include optic disc edema, optic disc pallor, and retinal hemorrhages, among others.^2,3,5-7^ While the full spectrum of fundus pathology may exceed the primary scope of neurology, exposure to a broader range of presentations can strengthen residents' examination skills and confidence in identifying critical deviations from normal. Neurology residents may also benefit from rotating through the ophthalmology inpatient consult service, which may offer exposure to a higher density and range of pathology.^32^ Incorporating virtual reality simulators, such as the Eyesi Direct Ophthalmoscope Simulator, may also enhance residency training and broaden residents' experience with various fundus presentations. This technology allows for the use of a simulated handheld ophthalmoscope to examine an artificial human face model, with adjustable physiologic and pathologic features of the fundus, providing a realistic training environment.^8,15^ Moreover, beyond their role in teaching, simulators may also serve as an adjunct objective tool for assessing resident skill acquisition.^8^ Barriers to implementation of this equipment in training are related to high cost and necessity for trained staff.^33^

To reach the ultimate goal of training neurologists who are competent in performing DO, interpreting the findings, and appropriately adjusting patient diagnosis and management, several objectives must be fulfilled. First, a specific definition of the threshold for competence should be established and adopted at the national level. In conjunction, a standardized assessment to determine competence in DO should be developed and validated in the context of neurologists entering clinical practice. Finally, a feasible, accessible, and effective DO curriculum must be developed and disseminated. It is important to note that while implementation of our current curriculum achieved substantial improvement on subjective and objective metrics, there is still room for improvement. For example, a sizable portion of residents remained unconfident in key components of the DO examination, including finding the optic disc (40.0%), finding retinal blood vessels (70.0%), and recognizing pathology (90.0%) (Figure 2). Further curricular innovation and iterative reform are required to investigate additional educational strategies that boost competence and self-efficacy in DO. As a pilot study, this work serves as an initial step toward refining, scaling, and adapting the curriculum for eventual multicenter implementation across varied training programs.

With technological advancements such as nonmydriatic fundus photography, the utility of DO has been questioned in recent years.^20,21^ The Fundus Photography vs Ophthalmoscopy Trial Outcomes in the Emergency Department (FOTO-ED) study demonstrated that nonmydriatic fundus imaging offers higher diagnostic accuracy and is more user-friendly than conventional DO.^34,35^ In addition, smartphone fundoscopy has shown promise, outperforming both nonmydriatic fundus imaging and DO in diagnostic accuracy when used by medical students.^36^ Despite these advancements, the limited availability and high cost of novel fundus cameras and smartphone fundoscopy systems relative to DO make them far less feasible for widespread use, particularly in under-resourced settings.^21^ Smartphone-based fundoscopy hardware may be less expensive ($400–$600) compared with direct ophthalmoscopes ($500–$1,000), yet it represents a new capital investment, given its limited adoption and reliance on compatible devices and software to optimize imaging.^9,37,38^ By contrast, direct ophthalmoscopes are already standard equipment in most hospitals, with primarily routine maintenance costs.^5^ Nonmydriatic cameras represent an even greater expense, typically exceeding $3,000, and are not routinely available in clinical practice.^9,21,39^ Only 4.3% of residents reported using nonmydriatic fundus imaging and 2.9% had used smartphone-based imaging, compared with 65.7% who used conventional direct ophthalmoscopes and 87.1% who used PanOptic direct ophthalmoscopes.^9^ DO is likely to remain an essential adjunct in clinical practice because of its real-time diagnostic ability and low cost, especially in regions where acquiring funding for equipment and training is more challenging.^39^ Ultimately, selecting the appropriate fundus visualization method requires balancing examiner comfort, availability, and clinical setting, with DO remaining a practical option before alternative technologies become more accessible.

Our study had several sources of variability and limitations that may affect generalizability. While our curriculum emphasized spaced practice, an approach shown to enhance skill retention, the longitudinal model may not be feasible across all programs because of scheduling and logistical constraints.^40-42^ Session timing depended on both preceptor and resident availability, creating variability in practice frequency. Although the small sample size limited formal comparisons between institutions, UCLA residents' higher post-rotation scores may reflect both greater access to PanOptic ophthalmoscopes—which offer a wider field of view and easier usability—and more frequent session attendance.^43,44^ Attendance was tracked by resident name, but to maintain anonymity, questionnaires and assessments used unique identifiers, preventing linkage between performance data and session attendance. Variability in preceptor backgrounds and clinical settings may have further contributed to performance differences. UCSF residents saw approximately 3 patients per session, with 60.0% of well examinations and 40.0% of pathology, including optic disc drusen, optic atrophy, papilledema, and glaucoma. UCLA residents saw approximately 6 patients per session, with 33.3% of well examinations and 66.7% of pathology, including optic neuritis, idiopathic intracranial hypertension, cranial nerve palsies, and neurofibromatosis type 1. The small sample size and self-selection bias—because residents who chose to participate may have been more motivated to improve their DO skills, potentially influencing their subjective responses and objective performance—pose additional limitations to the study's generalizability. The study was also limited to Kirkpatrick level 2 outcomes, as it primarily measured immediate changes in confidence and technical skills rather than changes in clinical practice outside the structured rotation.^23^ The longitudinal design of the curriculum was intended to reinforce skill retention over time; however, it remains unknown whether the skills acquired are sustained beyond the rotation period or subject to skill decay in the absence of continued practice. Future research should address these limitations by expanding sample sizes, standardizing curriculum elements and equipment, and incorporating longitudinal follow-up to better evaluate curriculum effectiveness and applicability.

In conclusion, the implementation of a dedicated optometry or general ophthalmology rotation for neurology residents has proven to be an effective strategy for enhancing both competency and confidence in DO. By providing structured, hands-on practice opportunities coupled with feedback from experienced clinicians, the curriculum addressed a critical gap in neurology training. While outcomes suggest meaningful progress, continued refinement is needed to support more consistent skill development across all learners. As a pilot study, this model offers a practical starting point for improving DO skills in neurology training, particularly in programs without access to neuro-ophthalmology rotations. Ultimately, this curriculum represents an early innovation in DO training that is accessible and adaptable, offering a promising foundation for improving DO competency and guiding broader implementation.

## Glossary

ABPN: American Board of Psychiatry and Neurology
ACGME: Accreditation Council for Graduate Medical Education
DO: Direct ophthalmoscopy
FOTO-ED: Fundus Photography vs Ophthalmoscopy Trial Outcomes in the Emergency Department
IRB: Institutional Review Board
PD: Program director
PGY: Postgraduate year
UCLA: University of California, Los Angeles
UCSF: University of California, San Francisco
